# Supplementary material for: Bright ligand-activatable fluorescent protein for high-quality multicolor live-cell super-resolution microscopy
Source: Nat Commun. 2020 Jan 14;11:273. doi: 10.1038/s41467-019-14067-4 (PMC6959352; doi:10.1038/s41467-019-14067-4)
Supplement: Supplementary file 1 — Supplementary Information [file 41467_2019_14067_MOESM1_ESM.pdf]

Bright Ligand-activatable Fluorescent Protein for High-quality Multicolor Live-cell Super-resolution Microscopy

Jiwoong Kwon[‡,1], Jong-Seok Park[‡,2], Minsu Kang[1,3], Soobin Choi[4], Jumi Park[5], Gyeong Tae Kim[6], Changwook Lee[5], Sangwon Cha[4], Hyun-Woo Rhee*[2,§] and Sang-Hee Shim*[1,3]

[1]Center for Molecular Spectroscopy and Dynamics, Institute for Basic Science (IBS), Seoul 02841, Republic of Korea.

[2]Department of Chemistry, Ulsan National Institute of Science and Technology (UNIST), Ulsan 44919, Republic of Korea.

[3]Department of Chemistry, Korea University, Seoul 02841, Republic of Korea.

[4]Department of Chemistry, Hankuk University of Foreign Studies, Yongin 17035, Republic of Korea.

[5]Department of Biological Sciences, Ulsan National Institute of Science and Technology (UNIST), Ulsan 44919, Republic of Korea.

[6]Department of Biochemical Engineering, Ulsan National Institute of Science and Technology (UNIST), Ulsan 44919, Republic of Korea.

[‡]These authors contributed equally to this work.

[§]Present address: Department of Chemistry, Seoul National University, Seoul 08826, Republic of Korea.

*Correspondence and requests for materials should be addressed to H.-W.L. (email: rheehw@snu.ac.kr) and/or to S.-H.S. (email: sangheeshim@korea.ac.kr).

Supplementary Note

1. Derivation of Analytical Solutions for On-switching Kinetics

The on-switching rate (k_{on}) was extracted from the recovery of fluorescence intensity after complete bleaching (Fig. 2e). Since UnaG can have a number of distinct conformations including two fluorescent states, the fluorescence intensity did not increase in a simple exponential manner. Therefore, we built a model including multiple fluorescent and nonfluorescent states for the fluorescence switching of UnaG to find the on-switching rate constants for the fluorescence recovery (Supplementary Figure 2a).

In our model, two fluorescent conformations of UnaG (holoUnaG1 and holoUnaG2) are bleached by absorption of blue light *via* photo-oxidation of bilirubin (BR) bound on UnaG, and produce a nonfluorescent product (damUnaG) that is a complex of UnaG protein and photo-oxidized BR (OxBR). Dissociation of OxBR leaves apoUnaG alone with empty binding pocket for other fresh BRs. Finally, apoUnaG is transformed to the holoUnaG1 upon the binding of fresh BR to recover the fluorescence, and holoUnaG1 spontaneously transits to holoUnaG2.

For the fluorescence recovery, after all of holoUnaGs were photo-oxidized, the various species of UnaG undergo reactions as below (and Supplementary Figure 2a) until they reach to the steady state.

$$\text{damUnaG} \xrightarrow{k_{\text{da}}} \text{apoUnaG} + \text{OxBR} \quad (1)$$

$$\text{apoUnaG} + \text{BR} \xrightarrow{k_{\text{ah}}} \text{holoUnaG1} \quad (2)$$

$$\text{holoUnaG1} \xrightarrow{k_{\text{h}}} \text{holoUnaG2} \quad (3)$$

$$\text{holoUnaG2} \xrightarrow{k'_{\text{h}}} \text{holoUnaG1} \quad (4)$$

Here, k_{da} is the rate constant of the dissociation of OxBRs from damUnaG, and k_{ah} is the rate constant of the binding of the BR to apoUnaG. k_h and k_h' are the rate constants of spontaneous transitions between holoUnaG1 and holoUnaG2. A graphical scheme for all the possible reactions in Supplementary Equation 1-4 is presented in Supplementary Figure 2a. Then, the rates of formation/dissociation of each population can be described as follows.

$$\frac{d}{dt}[\text{damUnaG}] = -k_{\text{da}}[\text{damUnaG}] \quad (5)$$

$$\frac{d}{dt}[\text{apoUnaG}] = k_{\text{da}}[\text{damUnaG}] - k_{\text{ah}}[\text{BR}][\text{apoUnaG}] \quad (6)$$

$$\frac{d}{dt}[\text{holoUnaG1}] = k_{\text{ah}}[\text{BR}][\text{apoUnaG}] - k_{\text{h}}[\text{holoUnaG1}] + k'_{\text{h}}[\text{holoUnaG2}] \quad (7)$$

$$\frac{d}{dt}[\text{holoUnaG2}] = k_{\text{h}}[\text{holoUnaG1}] - k'_{\text{h}}[\text{holoUnaG2}] \quad (8)$$

By assuming a complete bleaching of fluorescent species (i.e. $[\text{damUnaG}]_0 = 1$), and the amount of BR is much more than the total amounts of UnaG species (i.e. $[\text{BR}]_t = [\text{BR}]_0$), we can find the analytical solutions for the time-dependent populations of each species as follows.

$$[\text{damUnaG}]_t = exp(-k_{\text{da}}t) \quad (9)$$

$$[\text{apoUnaG}]_t = \frac{k_{\text{da}}}{k'_{\text{ah}}-k_{\text{da}}} exp(-k_{\text{da}}t) - \frac{k_{\text{da}}}{k'_{\text{ah}}-k_{\text{da}}} exp(-k'_{\text{ah}}t) \quad (10)$$

$$[\text{holoUnaG1}]_t = \frac{k'_{\text{h}}}{k_{\text{h}}+k'_{\text{h}}} + \frac{k'_{\text{ah}}(k'_{\text{h}}-k_{\text{da}})}{(k'_{\text{ah}}-k_{\text{da}})(k_{\text{da}}-k_{\text{h}}-k'_{\text{h}})} exp(-k_{\text{da}}t) - \frac{k_{\text{da}}(k'_{\text{h}}-k'_{\text{ah}})}{(k'_{\text{ah}}-k_{\text{da}})(k'_{\text{ah}}-k_{\text{h}}-k'_{\text{h}})} exp(-k'_{\text{ah}}t) + \frac{k_{\text{da}}k'_{\text{ah}}k_{\text{h}}}{(k_{\text{h}}+k'_{\text{h}})(k_{\text{da}}-k_{\text{h}}-k'_{\text{h}})(k'_{\text{ah}}-k_{\text{h}}-k'_{\text{h}})} exp\{-(k_{\text{h}}+k'_{\text{h}})t\} \quad (11)$$

$$[\text{holoUnaG2}]_t = \frac{k'_{\text{h}}}{k_{\text{h}}+k'_{\text{h}}} + \frac{k'_{\text{ah}}k_{\text{h}}}{(k'_{\text{ah}}-k_{\text{da}})(k_{\text{da}}-k_{\text{h}}-k'_{\text{h}})} exp(-k_{\text{da}}t) - \frac{k_{\text{da}}k_{\text{h}}}{(k'_{\text{ah}}-k_{\text{da}})(k'_{\text{ah}}-k_{\text{h}}-k'_{\text{h}})} exp(-k'_{\text{ah}}t) - \frac{k_{\text{da}}k'_{\text{ah}}k_{\text{h}}}{(k_{\text{h}}+k'_{\text{h}})(k_{\text{da}}-k_{\text{h}}-k'_{\text{h}})(k'_{\text{ah}}-k_{\text{h}}-k'_{\text{h}})} exp\{-(k_{\text{h}}+k'_{\text{h}})t\} \quad (12)$$

Here, k_{ah}' is the binding constant of BR to the apoUnaG at a certain concentration of BR (i.e. $k_{\text{ah}}' = k_{\text{ah}}$[BR]), and it varies under different concentration of BR. Only the holoUnaG species are fluorescent, and holoUnaG2 is ~3.9 times brighter than holoUnaG1. Therefore, the fluorescence intensity that we observed is $[\text{holoUnaG1}]_t + 3.9[\text{holoUnaG2}]_t$. Finally, we can normalize the fluorescence intensity to the steady state, where the holoUnaG1 and holoUnaG2 exist with a ratio of 6:4, by dividing the fluorescence intensity with a factor of 2.16.

$$F.I._{norm} = \frac{1}{2.16}\{[\text{holoUnaG1}]_t + 3.9[\text{holoUnaG2}]_t\} = \frac{1}{2.16}\left[\frac{3.9k_{\text{h}}+k'_{\text{h}}}{k_{\text{h}}+k'_{\text{h}}} + \frac{k'_{\text{ah}}(3.9k_{\text{h}}+k'_{\text{h}}-k_{\text{da}})}{(k'_{\text{ah}}-k_{\text{da}})(k_{\text{da}}-k_{\text{h}}-k'_{\text{h}})} exp(-k_{\text{da}}t) - \frac{k_{\text{da}}(3.9k_{\text{h}}+k'_{\text{h}}-k'_{\text{ah}})}{(k'_{\text{ah}}-k_{\text{da}})(k'_{\text{ah}}-k_{\text{h}}-k'_{\text{h}})} exp(-k'_{\text{ah}}t) - \frac{2.9k_{\text{da}}k'_{\text{ah}}k_{\text{h}}}{(k_{\text{h}}+k'_{\text{h}})(k_{\text{da}}-k_{\text{h}}-k'_{\text{h}})(k'_{\text{ah}}-k_{\text{h}}-k'_{\text{h}})} exp\{-(k_{\text{h}}+k'_{\text{h}})t\}\right] \quad (13)$$

In Supplementary Figure 2b-d, we simulated the fluorescence recovery for various dissociation rates of OxBR by using Supplementary Equation 13 and the rate constants obtained from our results at [BR] = 0.2-1.0 μM conditions. Note that the fluorescence recovery becomes a bi-exponential growth if the k_{da} is much larger than 1 s^{-1} at the simulated time scale and resolution (and also at the experimental time scale and resolution), whereas a sigmoidal-like behavior is observed with slower dissociation rate. In formal case, k_{da} can be treated as the infinite, and Supplementary Equation 13 is reduced as follow.

$$F.I._{norm} = \frac{1}{2.16}\left[\frac{3.9k_{\text{h}}+k'_{\text{h}}}{k_{\text{h}}+k'_{\text{h}}} + \frac{3.9k_{\text{h}}+k'_{\text{h}}-k'_{\text{ah}}}{(k'_{\text{ah}}-k_{\text{h}}-k'_{\text{h}})} exp(-k'_{\text{ah}}t) - \frac{2.9k'_{\text{ah}}k_{\text{h}}}{(k_{\text{h}}+k'_{\text{h}})(k'_{\text{ah}}-k_{\text{h}}-k'_{\text{h}})} exp\{-(k_{\text{h}}+k'_{\text{h}})t\}\right] \quad (14)$$

Supplementary Figure 2e describes Supplementary Equation 14 at [BR] = 0.2-1.0 μM conditions, and shows no significant difference to Supplementary Figure 2d. Because our experimental results showed bi-exponential growth for the fluorescence recovery (Fig. 2e), we used Supplementary Equation 14 to fit the experimental results to figure out the on-switching rate constants ($k_{\text{on}} = k_{\text{ah}}'$) by assuming that the dissociation rate of OxBR is much faster than 1 s^{-1} and is hard to precisely measure with our time resolution (1 s).

Supplementary Table 1 Information for gene constructs used in this study.

Name	Features	Promotor/ Vector	Forward Primer/ Reverse Primer	Notes
UnaG-Sec61β	*NotI*-UnaG-Flag-*EcoRI*-Sec61β-Stop-*XhoI*	CMV/ pCDNA3	tagtaggaattcatgcctggtccgacccc/tagtagctcgagtcacgaacgagtgtacttgcccca	Sec61β (NM_006808)<br>Flag: DYKDDDDK
Vim-UnaG	*HindIII*-Vim-Linker-Flag-*NheI*-UnaG-Stop-*XhoI*	CMV/ pCDNA5	tagtagaagcttatgtccaccaggtccgtgtc/ggatccttagatccaccagatccaccagattcaaggtcatcgtgatgctgaga	pVim-mMaple3 was a gift from Xiaowei Zhuang (Harvard Univ.)<br>Linker: SGGSGGS
PMP70-UnaG	*KpnI*-PMP70-*BamHI*-*NheI*-Flag-UnaG-Stop-*NotI*	CMV/ pCDNA5	tagtaggctagcagactacaaggatgacgacgataagatggttgaaaaatttgttggtacgtgga/tagtaggcggccgctcattattcggttgcacgccggta	PMP70 (NM_002858.3)
UnaG-LaminA/C	*NotI*-UnaG-Flag-*EcoRI*-LaminA/C-Stop-*XhoI*	CMV/ pCDNA3	tagtaggaattcatggagaccccgtcccagcgg/tagtagctcgagtcattacatgatgctgcagttctggggg	LaminA/C was a gift from Bo Hunag (UCSF)
Mito-UnaG	*NotI*-Mito-*BamHI*-UnaG-Flag-Stop-*XhoI*	CMV/ pCDNA3	tagtagggatccaatggttgaaaaatttgttggtacgtggaaaatagcg/tagtagctcgagtcattacttatcgtcgtcatccttgtagtcttcggttgcacgccggtacg	Mitochondrial Matrix sequence: MLATRVFSLVGKRAISTSVCVRAH
UnaG-CLC	*NotI*-UnaG-Flag-*EcoRI*-CLC-Stop-*XhoI*	CMV/ pCDNA3	tagtaggaattcatggccgagttggatccattcg/tagtagctcgagtcagtgcaccaggggggcc	YFP-Clathrin was a gift from Xiaowei Zhuang (Addgene plasmid # 20921)
UnaG-HaloTag-CLC	*NotI*-UnaG-Flag-*EcoRI*-HaloTag-*AflII*-CLC-Stop-*XhoI*	CMV/ pCDNA3	tagtaggaattcatggcagaaatcggtactggctttcca/tagtagctcgagtcagtgcaccaggggggcc	HaloTag PDB ID: 4KAF
UnaG-APtag	*NotI*-UnaG-Flag-*EcoRI*-APtag-Stop-*XhoI*	CMV/ pCDNA3	aattcgggtccgggttgaatgatattttcgaagcacagaaaattgaatggcatgagtgac/tcgagtcactcatgccattcaattttctgtgcttcgaaaatatcattcaacccggacccg	APtag: GLNDIFEAQKIEWHE
UnaG-mCherry	*NotI*-UnaG-Flag-*EcoRI*-mCherry-*BamHI*-His6-Stop-*XhoI*	T7/ pET21a	tagtaggcggccgcatggttgaaaaatttgttggtacgtggaaaatagcg/tagtagggatcccttgtacagctcgtccatgccg	His6: HHHHHH
TfR-PAmCherry	*BglII*-TfR-*AgeI*-PAmCherry1-Stop-*NotI*	CMV/ pEGFP	N.A.[a]	pTfR-PAmCherry1 was a gift from Vladislav Verkhusha (Addgene plasmid # 31948)
Vim-mMaple3	*NheI*-Vim-Linker-mMaple3-Stop-*NotI*	CMV/ pmEos2	N.A.[a]	pVim-mMaple3 was a gift from Xiaowei Zhuang (Harvard Univ.)
Vim-EGFP	*HindIII*-Vim-Linker-EGFP-Stop-*NotI*	CMV/ pEGFP	N.A.[a]	EGFP-Vimentin-7 was a gift from Michael Davidson (Addgene plasmid # 56439)
HaloTag-TM	ss-HA-*BglII*-Flag-HaloTag-*SalI*-TM	CMV/ pCDNA3	tagtagagatctatggcagaaatcggtactggctttccat/tagtaggtcgacagtggttggctcgccggaaatc	HA epitope tag: PYDVPDYA<br>TM: Transmembrane domain
BirA	*XbaI*-BirA-*HindIII*-*XhoI*-His6-Stop	T7/ pET28a	N.A.[a]	BirA PDB ID: 1BIB
pSNAP-FtnA	*AgeI*-SNAPf-*NheI*-FtnA-Stop-*BamHI*	CMV/ PAtagRFP-actin	N.A.[a]	pSNAP-FtnA was a gift from Mike Heilemann (Addgene plasmid # 98282)

UnaG-FtnA	*AgeI*-UnaG-Flag-*NheI*-FtnA-Stop-*BamHI*	CMV/ PAtagRFP-actin	tagtaggaattcggaggatccgcaggaatgctgaaaccagaaatgattgaaaaa/tagtagctcgagttagttttgtgtgtcgagggtagagagtt	pSNAP in pSNAP-FtnA was replaced with UnaG

[a] Not available since we directly used the plasmid without cloning.

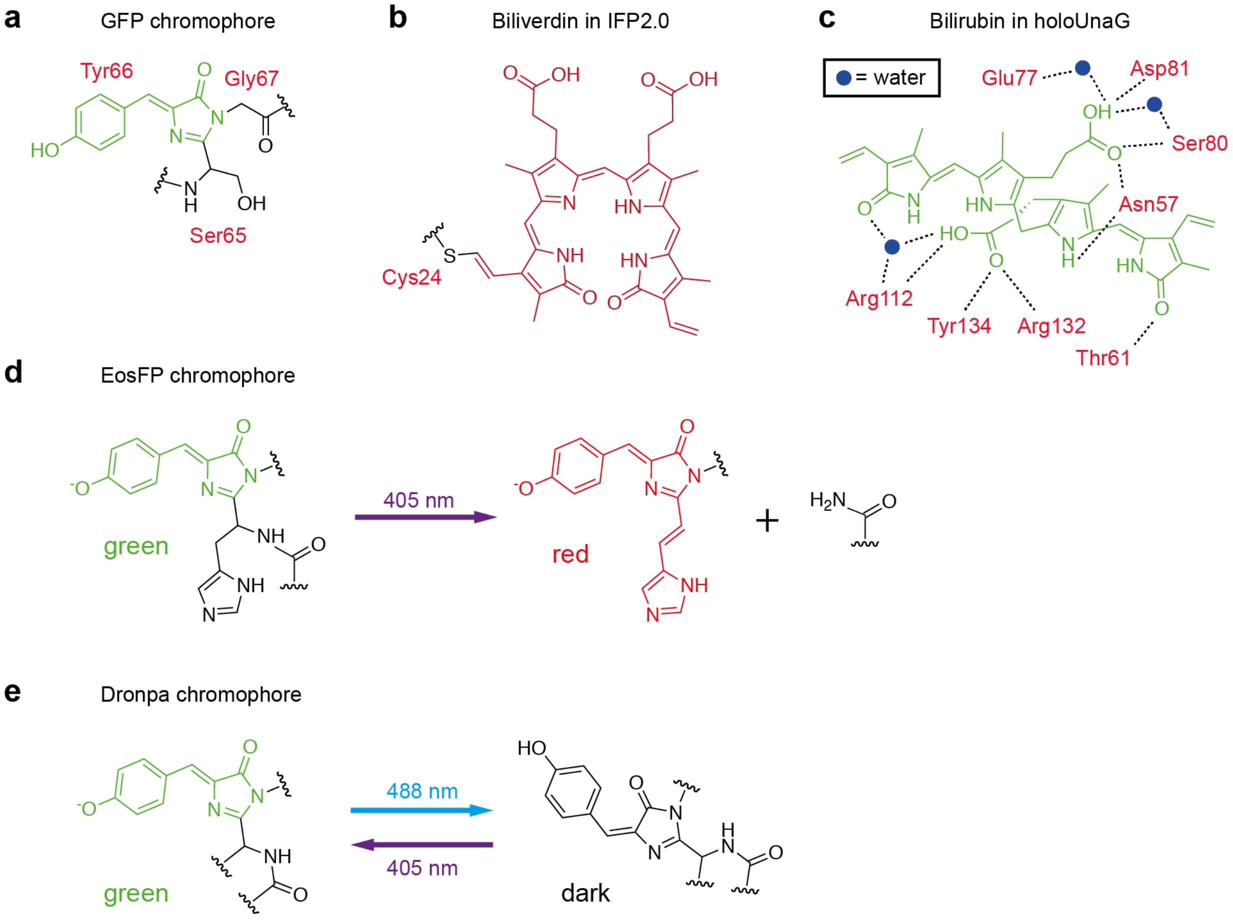

Supplementary Figure 1 Chromophore structures of fluorescent proteins. **a** GFP chromophore produced from Ser65, Gly67 and Tyr66 of *Aequorea* GFP. **b** Biliverdin structure covalently linked to Cys24 of IFP2.0. **c** UnaG found in eel with noncovalently bound fluorogenic ligand of bilirubin. The binding cavity of UnaG forms many hydrogen bonds (dotted lines) between amino acids (red texts), water molecules (blue dots) and bilirubin (green molecule). **d**,**e** Chromophores of photoswitching fluorescent proteins used in single-molecule localization microscopy. **d** Photoconvertible FPs such as EosFP convert reversibly from a green fluorescent form to a red fluorescent state upon illumination of near ultraviolet light. The red-shift is caused by the extension of the conjugation of the chromophore after the cleavage of the peptide backbone. **e** Photoswitching FPs such as Dronpa undergo reversible *cis*-*trans* isomerization. The *cis* form is fluorescent and the *trans* form is nonfluorescent.

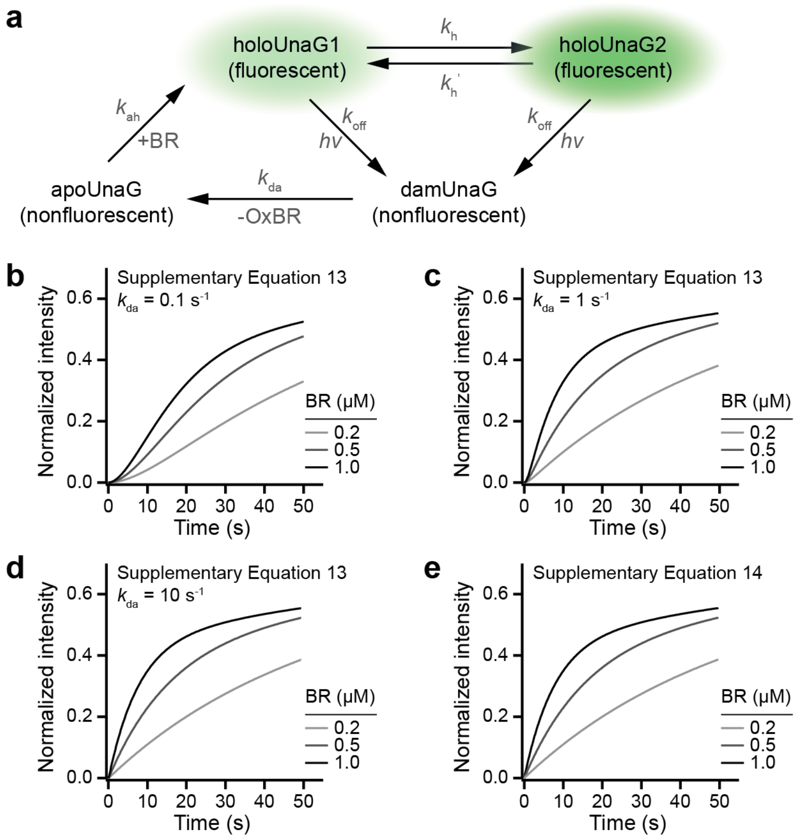

Supplementary Figure 2 Derivation of analytical solution for the fluorescence recovery of UnaG. **a** Switching model with all possible states of UnaG. We considered two nonfluorescent UnaGs, damUnaG (complex of OxBR and UnaG) and apoUnaG (UnaG protein alone), as well as two fluorescent forms of UnaG, holoUnaG1 and holoUnaG2. Here the rate constants k_h, k_h' are related to the spontaneous transition between two conformations of holoUnaG, where k_{off}, k_{da}, k_{ah} are the rate constants for the photo-oxidation (from holoUnaGs to damUnaG, k_{off}), dissociation of OxBR (from damUnaG to apoUnaG, k_{da}) and binding of fresh BR (from apoUnaG to holoUnaG1, k_{ah}) of each species. **b**-**d** Simulation results for [BR]-dependent fluorescence recovery by using Supplementary Equation 13 in different rate constants for OxBR from damUnaG (k_{da} = 0.1, 1, 10 s^{-1} for **b**, **c** and **d**, respectively). If the dissociation rate is slow enough compared to the time resolution, the fluorescence recovery shows sigmoidal-like behavior (**b** and **c**). However, if the dissociation rate is too fast to observe the dissociation event with the time resolution, the fluorescence recovery can be approximated to the bi-exponential rising (**d**). **e** Simulation result for [BR]-dependent fluorescence recovery by using Supplementary Equation 14. Note that the pattern of the fluorescence recovery is almost identical to the results showed in **d**.

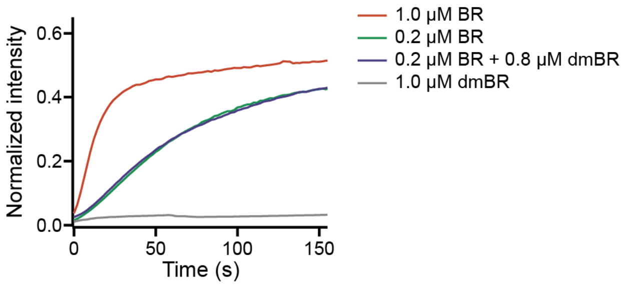

Supplementary Figure 3 Fluorescence recovery of photobleached holoUnaG in different buffer conditions. Photo-damaged BR (dmBR) was produced separately by illuminating a solution of BR with an intense 488-nm laser (120 mW) until the solution becomes transparent (~30 min). Note that photo-damaged BR (dmBR) could not recover the fluorescence of UnaG regardless of the amount and did not compete with undamaged bilirubin for binding with apoUnaG.

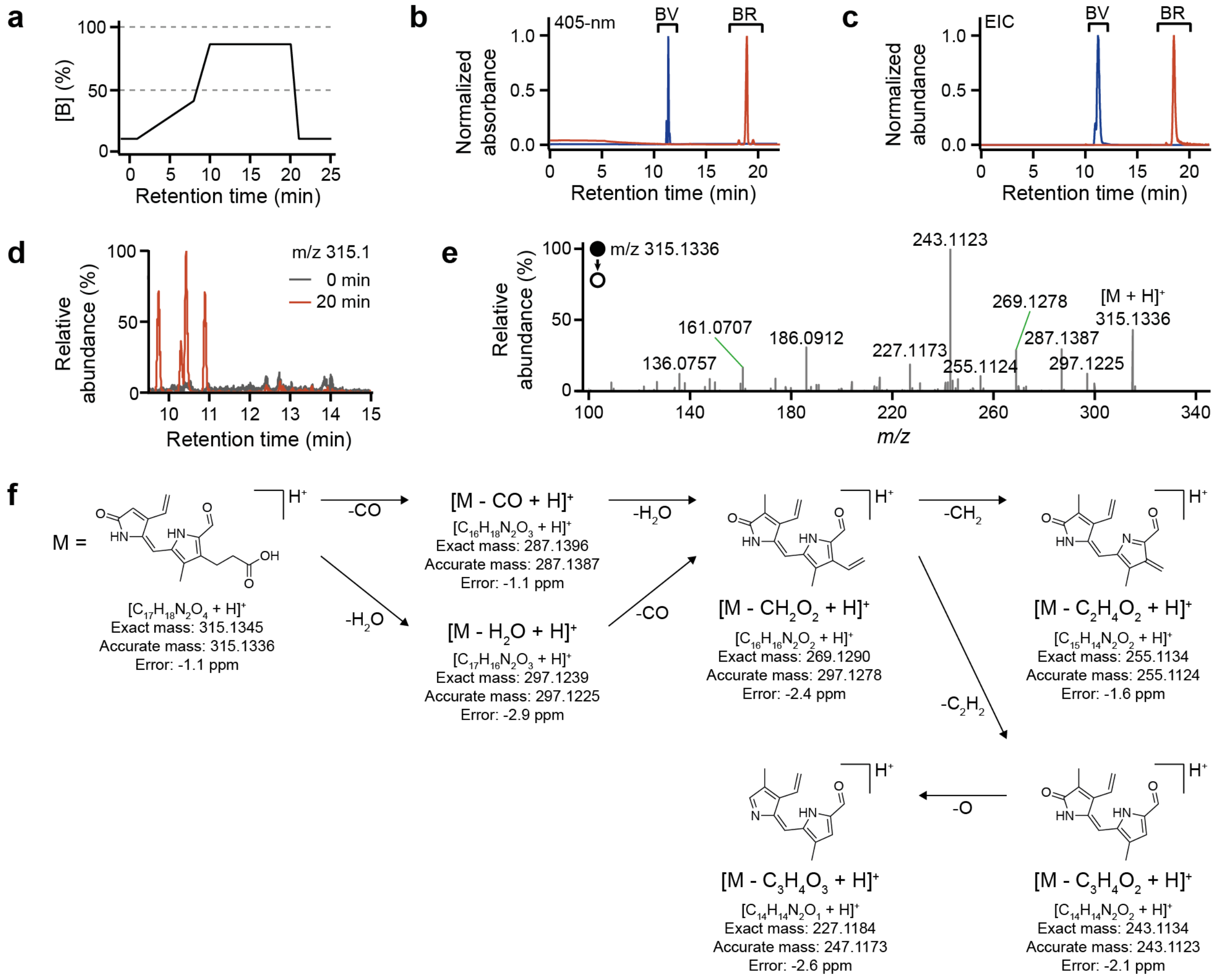

Supplementary Figure 4 LC-UV/vis and LC/MS analysis of the UnaG photo-oxidation products. **a** Solvent gradient profile used for both LC-UV/vis and LC/MS experiments. We used two mobile phases (A: 0.1% formic acid in 95/5 water/acetonitrile and B: 0.1% formic acid in acetonitrile) to gradually control the hydrophobicity of eluent. **b** UV/vis chromatogram at 405 nm and **c** Normalized extracted ion chromatogram (EIC) of standard samples of pure bilirubin (BR) and biliverdin (BV) under the same LC condition. BR and BV were clearly identified by using both of 405-nm absorbance and MS with identical retention times. **d** EIC of the protonated OxBR ion at *m/z* 315.1 for irradiation times of 0 min (grey) and 20 min (red), respectively. Similar to Fig. 3**a**, intensities of OxBR peaks were significantly increased with light irradiation in LC/MS analysis. **e** HRMS/MS spectrum of the ion corresponding to *m/z* 315.1336 (Fig. 3b). **f** Interpretation of fragment ions in panel **e** with possible chemical structures. Elemental compositions of fragment ions were determined with mass tolerance of 5 ppm. Mass errors between exact masses of determined compositions and accurate masses were calculated. P: propionic acid (-CH_2CH_2COOH); V: vinyl (-$CH=CH_2$).

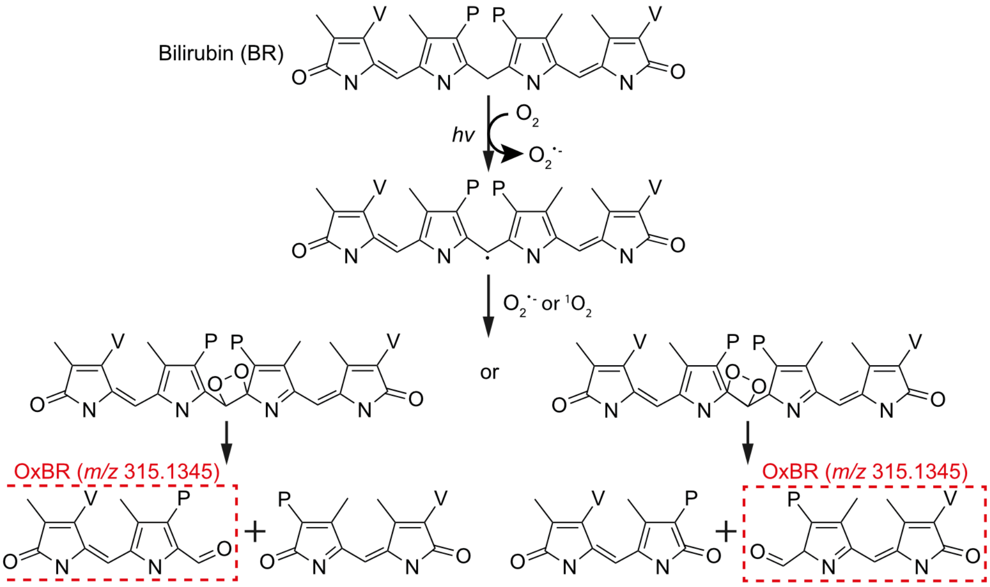

Supplementary Figure 5 Proposed reaction mechanism for the photo-oxidation of BR in holoUnaG to form OxBR in Fig. 3**b**. Light absorption facilitates the oxidation of BR with dissolved oxygen to produce radial species, which can be further oxidized by reacting with singlet oxygen or superoxide radical. The resultant four-membered ring with oxygen atoms can easily break down to form the ketone-containing products including OxBR in Fig. 3**b** (red dashed boxes). P: propionic acid (-CH_2CH_2COOH); V: vinyl (-$CH=CH_2$).

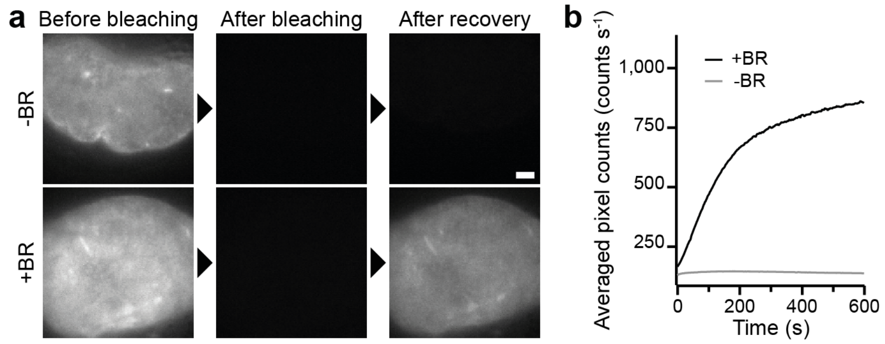

Supplementary Figure 6 Fluorescence recovery of UnaG in a fixed cell with and without external BR. **a** Representative fluorescence images for photoswitching of UnaG labeled on lamina. UnaG proteins were photobleached with ~300 W cm^{-2} of 488 nm laser for 10 s, and the following fluorescence recovery was measured after 10 min with 1 μM of external BR incubation. **b** Comparison of fluorescence recovery with and without external BR. The averaged pixel counts were extracted from 1-s snapshot of widefield images for 10 min. Scale bar: 2 μm.

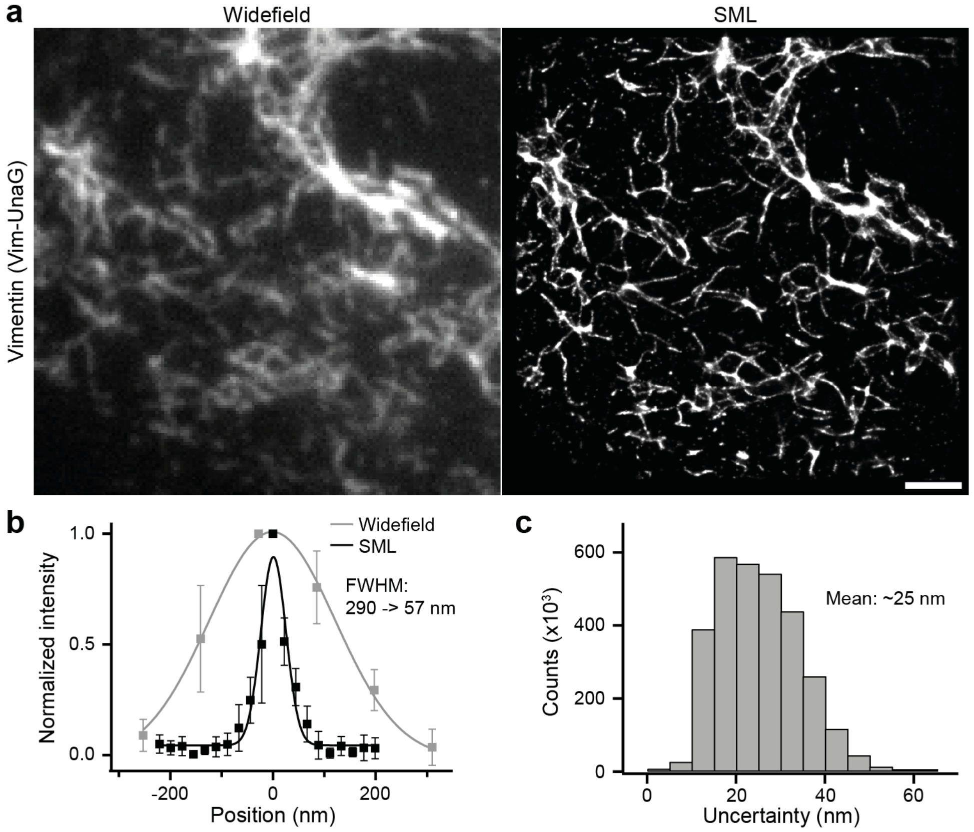

Supplementary Figure 7 SML imaging of UnaG on vimentin filaments. **a** Widefield (left) and super-resolution SML (right) images of UnaG-fused vimentin filaments in a fixed Cos7 cell. **b** Averaged intensity profiles of single vimentin filaments. **c** Resolution analysis of the reconstructed image by localization uncertainty analysis, which yielded ~25 nm of mean localization uncertainty. Scale bar: 2 μm. Error bars: standard deviations (n = 5).

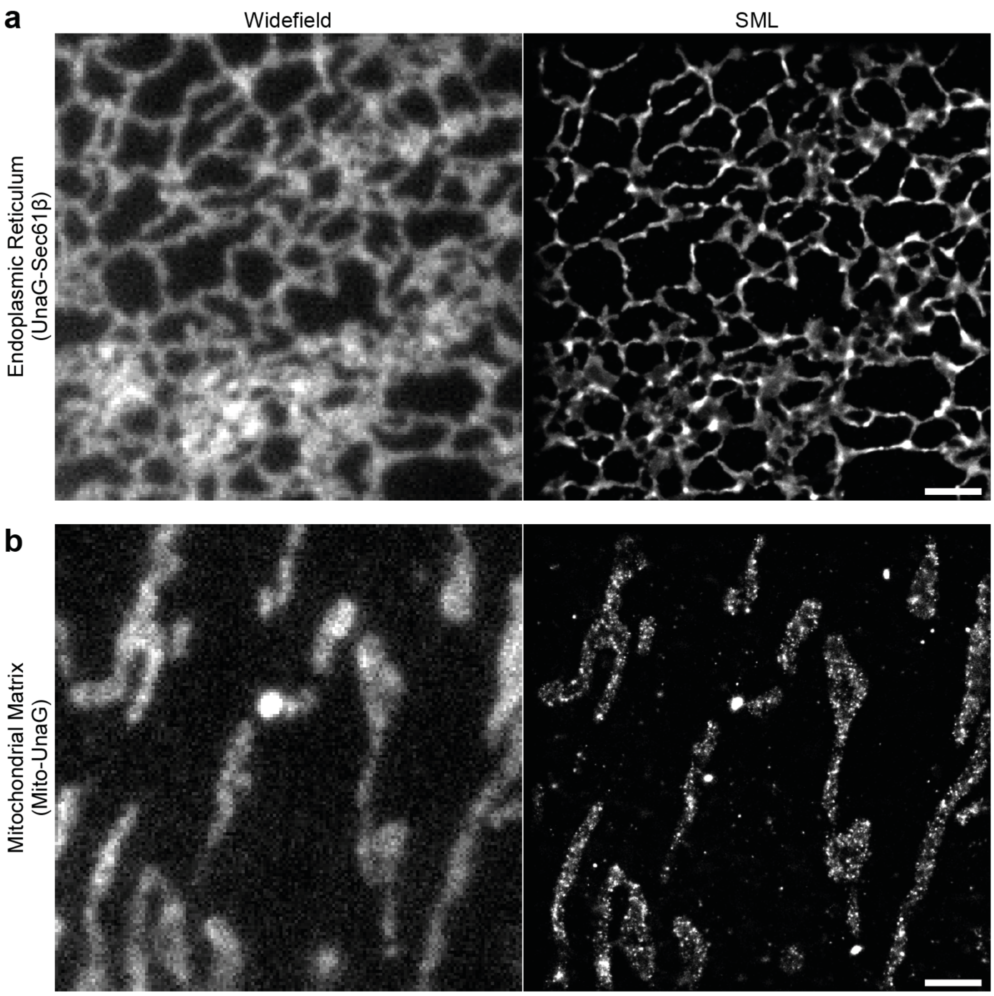

Supplementary Figure 8 SML imaging of UnaG on the endoplasmic reticulum (ER) and mitochondria. **a**,**b** Comparison of widefield (left) and SML (right) images in the full field of view for UnaG-fused to **a** Sec61β and **b** mitochondrial matrix targeting sequence. Scale bars: 2 μm.

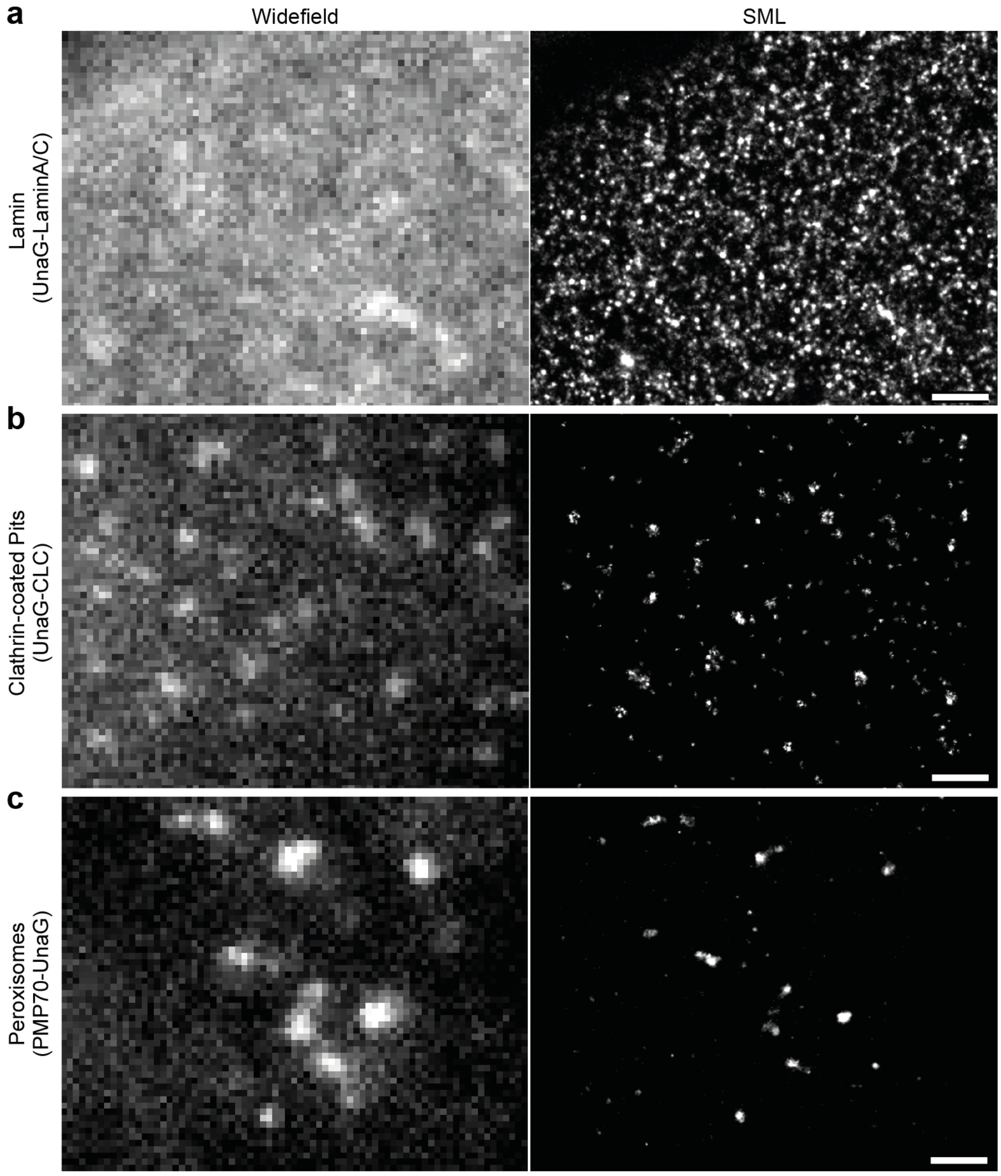

Supplementary Figure 9 SML imaging of UnaG on lamin, clathrin-coated pits and peroxisomes. **a**-**c** Comparison of widefield and SML images in full field of view for UnaG-fused to **a** LaminA/C, **b** clathrin light chains (CLC) and **c** peroxisomal protein, PMP70. Scale bars: 1 μm.

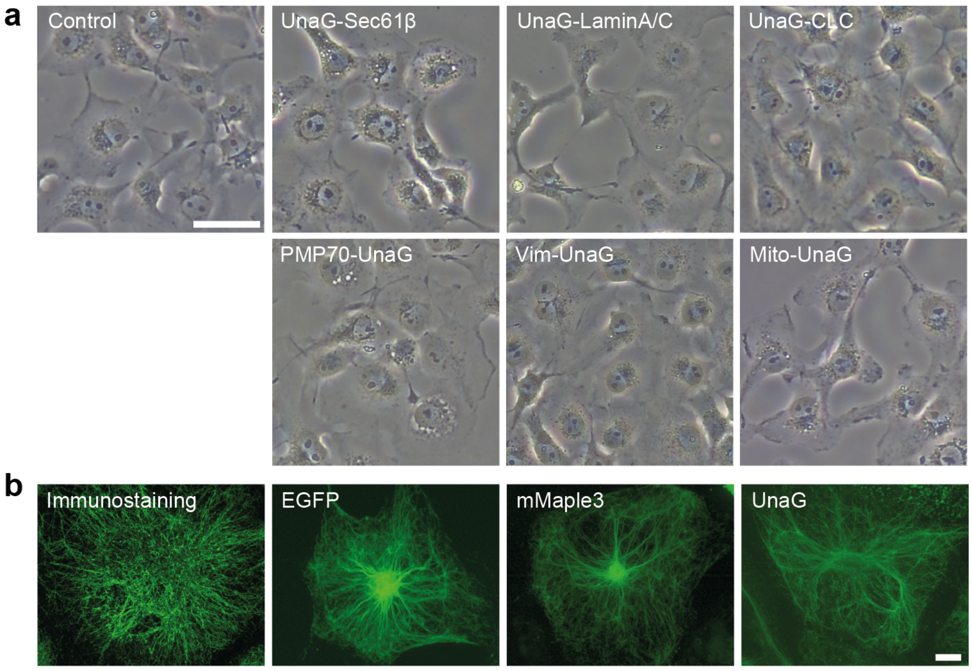

Supplementary Figure 10 Cell morphologies and vimentin structures upon UnaG expressions. **a** Brightfield images of live Cos7 cells expressing UnaG on various subcellular structures. No severe distortions were observed in the cell morphology regardless of the target structures. **b** Fluorescence confocal images of vimentin filaments labeled by Alexa Flour 488 (immunostaining), EGFP, mMaple3 and UnaG. The cells were scanned from the bottom to the top along the optical axis, and the images were projected with the maximum pixel intensities. UnaG-labeled vimentin showed mostly thin filaments observed in the immunostained cells, with slight bundling comparable to those observed in other FPs with minimal dimerization characters such as EGFP and mMaple3. Scale bars: 10 μm for **a**; 2 μm for **b**.

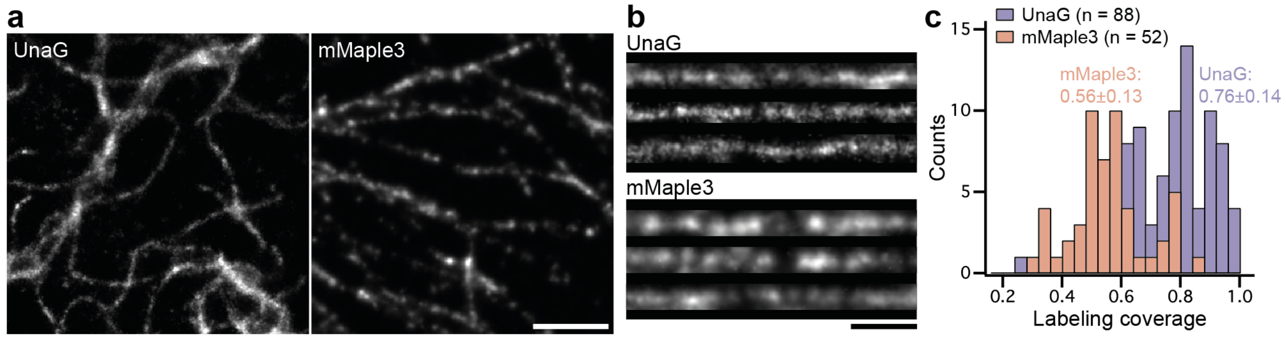

Supplementary Figure 11 Direct comparison of SML images and lengthwise labeling coverages of vimentin fibers labeled with UnaG and mMaple3. **a** SML images of vimentin fused with UnaG and mMaple3. **b** Traced and straightened vimentin fibers labeled with UnaG (top) and mMaple3 (bottom), obtained from **a** by using manual segmentation and straighten selection tools in ImageJ. **c** Lengthwise labeling coverage histograms obtained from 88 fibrils for UnaG and 52 fibrils for mMaple3 as exemplified in **b**. Scale bar: 1 μm for **a**; 500 nm for **b**. Error values (± values): standard deviations (n = indicated in **c**).

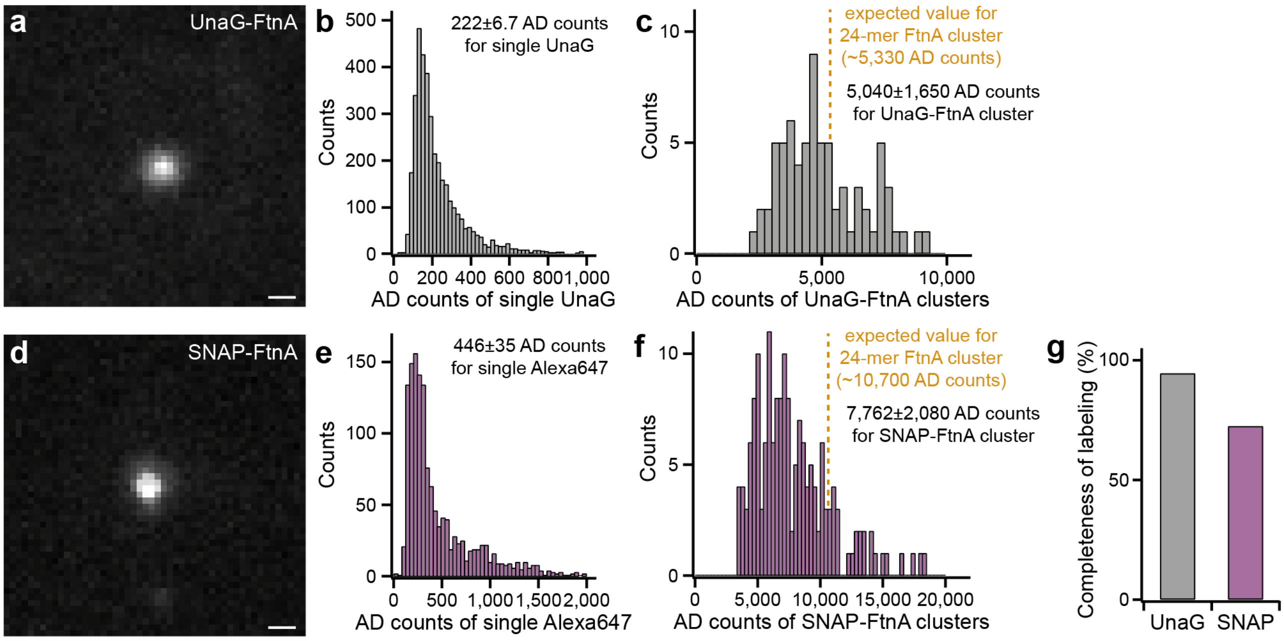

Supplementary Figure 12 Completeness of labeling for UnaG and SnapTag labeling system using FtnA-oligomer standard of 24 subunits. Completeness of labeling is calculated as the percentage of the mean value of FtnA-24mer fluorescence intensity in respect to the theoretical estimation from the average single-molecule intensity. **a** Representative image of FtnA-24mer labeled with UnaG. **b** Single-molecule photon number distribution of UnaG in measurement conditions, presented with the mean value. **c** Intensity distribution of FtnA-UnaG oligomers. The mean value was used to calculate the completeness of labeling in **g**. **d**-**f** Same data set with **a**-**c** for SnapTag labeling system. **g** Completeness of labeling of UnaG and SnapTag calculated by dividing the average counts of the cluster in **c** and **f** by 24 times the average count of single molecules in **b** and **e**, and they yielded 94.6% and 72.5% for UnaG and SnapTag, respectively. Scale bars: 500 nm. Error values (± values): standard deviations (n = 5 field of vies for **b** (>1,500 molecules per field); 73 clusters for **c**; 4 field of views for **e** (>1,500 molecules per field); 173 clusters for **f**).

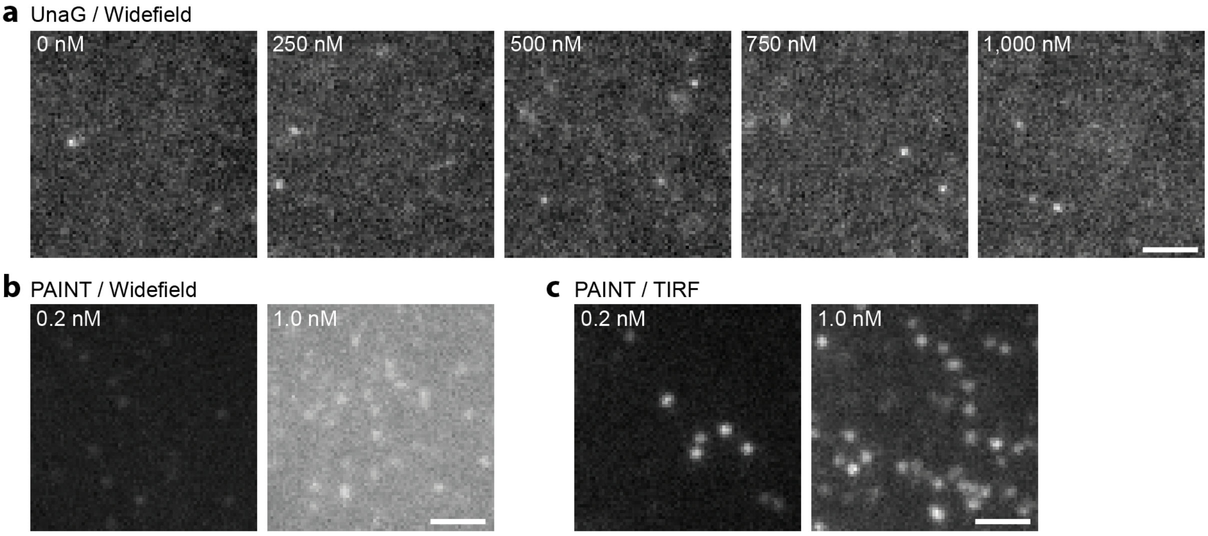

Supplementary Figure 13 Comparison of the raw single-molecule images of DNA-PAINT and UnaG under different illumination geometries. **a** In UnaG SML imaging, in which the unbound ligand of bilirubin is non-fluorescent, the backgrounds under Epi illumination geometry were virtually unchanged up to 1,000 nM concentration of exogenously supplemented bilirubin. **b** In PAINT imaging, even 1.0 nM of probe elevated the background significantly in Epi illumination. **c** Total internal reflection (TIR) illumination was required to decrease the fluorescence background of 1.0 nM PAINT probes. Scale bars: 2 μm.

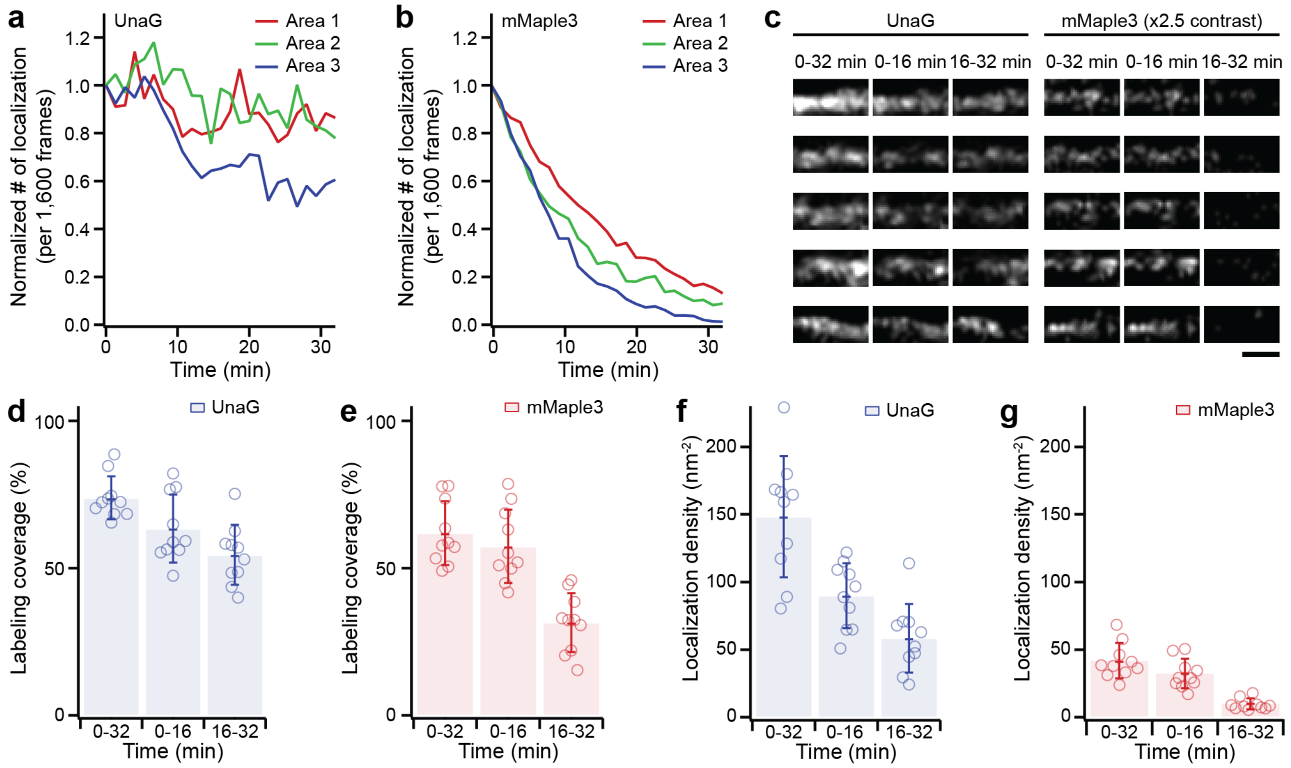

Supplementary Figure 14 Photobleaching resistance of SML imaging with UnaG in comparison to that with mMaple3. **a**,**b** Number of switching events (i.e. localizations) extracted from 32-min-long SML images of vimentin filaments labeled with UnaG (**a**) and mMaple3 (**b**). Raw data was recorded at an exposure time of 50 ms for 40,000 frames and analyzed with standard multi-emitter fitting algorithm in ThunderSTORM. The UnaG samples were supplemented with 1 µM bilirubin in solution. While the switching events of mMaple3 diminished due to irreversible photobleaching, UnaG continued to produce switched-on spots thanks to the fluorescence recovery by the fresh bilirubin ligands in solution. **c** Example SML images of individual vimentin filaments reconstructed with different time windows of the entire acquisition period (0-32 min), the first half (0-16 min) and the last half (16-32 min) of the period. UnaG molecules constantly switched on during the acquisition time, giving more continuous images as localization numbers accumulate. However, mMaple3 molecules quickly bleached and therefore longer acquisition time did not help to improve the image quality. **d**-**g** Average labeling coverages (**d**,**e)** and average localization densities (**f**,**g**) of individual vimentin filaments labeled by UnaG (**d**,**f)** and mMaple3 (**e**,**g**) with different acquisition times of the entire acquisition period (0-32 min), the first half (0-16 min) and the last half (16-32 min). Each data point on coverage and density was measured from 10 individual vimentin filaments. UnaG showed better labeling coverage and localization density than mMaple3 especially in the late time period (16-32 min). Scale bar: 200 nm. Error bars: standard deviations (n = 10 individual vimentin filaments for **d**-**g**).

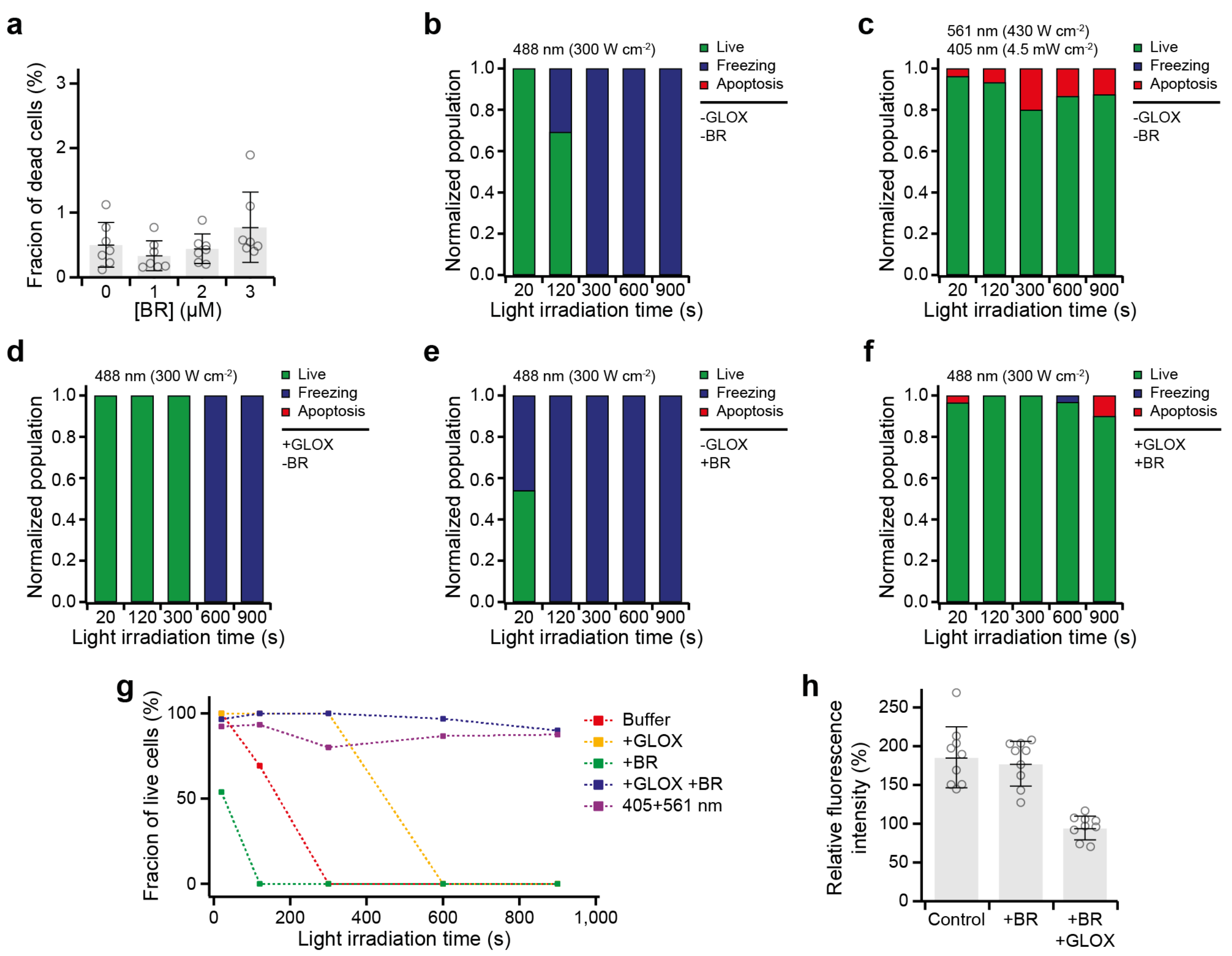

Supplementary Figure 15 Cellular toxicity tests for the live cell SML imaging of UnaG. **a** Cytotoxicity of external BR at high concentrations. The cells were incubated in BR containing media for 24 hours and the viability was measured by using a fluorescent live/dead assay, showing no substantial difference in the fraction of dead cells up to two-times higher concentration than experimental condition (1 μM). **b-f** Phototoxicity in various illumination and buffer conditions measured by observing the morphology and dynamics of cells after 3 hours from the light irradiation via brightfield imaging. 17-32 cells were observed per experiment and assigned as live, freezing and apoptotic by visual inspection. **b** Phototoxicity of intense 488-nm laser in live cell imaging buffer (phenol red-free DMEM supplemented with 75 mM of HEPES pH 8 and 2% of glucose). Cells were irradiated by 300 W cm^{-2} of 488-nm for the indicated irradiation times. The cells were rapidly frozen due to the photo-crosslinking effect of the intense blue light. **c** Phototoxicity of weak UV laser in live cell imaging buffer. The cells were irradiated by both of intense 561-nm laser (430 W cm^{-2}) and weak 405-nm laser (4.5 mW cm^{-2}), which are generally used for SML imaging of red fluorescent proteins with UV activation. The weak UV light induced apoptosis in some cells. **d** Phototoxicity of intense 488-nm laser in GLOX-containing live cell imaging buffer. GLOX slightly attenuated the phototoxicity of blue light. **e** Phototoxicity of intense 488-nm laser in BR-containing live cell imaging buffer. 1 μM of BR strongly accelerated the freezing effect, probably because of generation of radical species of BR after excitation by the 488-nm laser. **f** Phototoxicity of intense 488-nm laser in both of GLOX/BR-containing live cell imaging buffer. The cells survived under strong blue light irradiation, interestingly, when both of GLOX and BR are included in the buffer solution. One of possible mechanism is that the excess BR absorbs the blue light instead of the cells, and the GLOX helps to reduce the cytotoxic reactive oxygen species (ROS) by depleting the dissolved oxygen and also by depleting the hydrogen peroxide. In this condition, the dead cells showed both symptoms of freezing and apoptosis. **g** Summary for the

phototoxicity in various buffer compositions using the fraction of live cells. In our SML imaging conditions, most the cells can survive more than 3 hours under <15 min of strong 488-nm illumination. **h** ROS generation by the 300 W cm^{-2} of 488-nm laser irradiation in various buffer compositions measured by a fluorescent ROS indicator. A small area in a UnaG-expressed live Cos7 cell was illuminated by the blue light for 10 min, and the relative fluorescence intensity of the ROS indicator was measured from an unilluminated area of the same cell before and after illumination. While BR did not affect the ROS concentration, GLOX clearly decreased the concentration of ROS. Error bars: standard deviations (n = 7 individual measurements for **a**; 9 individual measurements for **h**)..

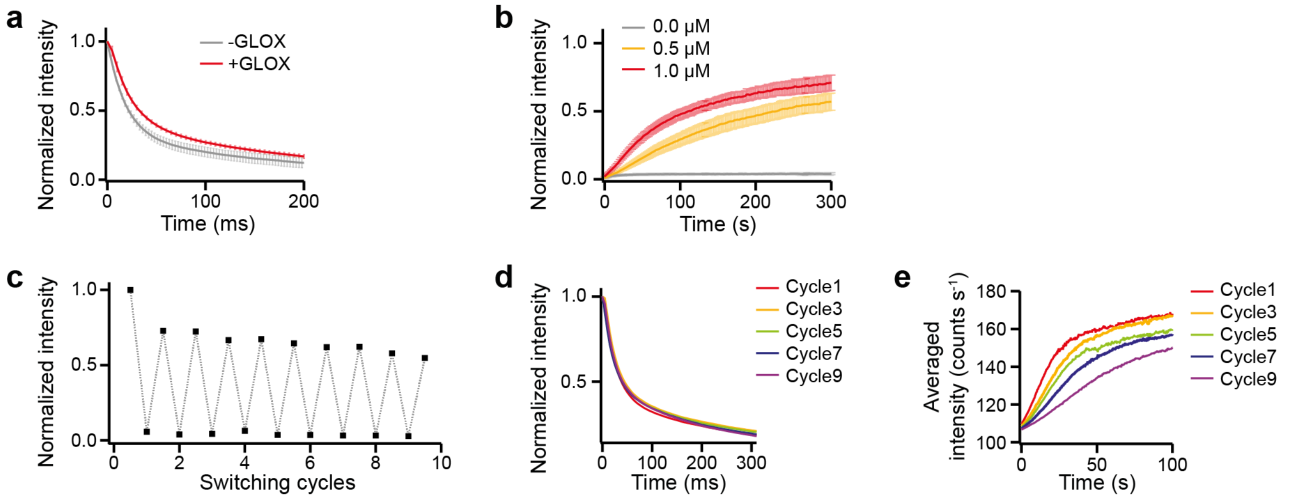

Supplementary Figure 16 Photo-switching properties of UnaG for live-cell SML imaging. **a** Effect of GLOX on off-switching rate of Sec61β-fused UnaG in a live cell. Although the difference was relatively smaller than the *in vitro* case in Fig. 2, GLOX can decrease the off-switching rate of UnaG inside a living cell. **b** Effect of external BR on on-switching rate of Sec61β-fused UnaG in a live cell. External BR clearly accelerates the on-switching rate. **c** Repetitive on- and off-switching events of UnaG in a live cell. Off-switching events were carried by 300 W cm^{-2} of 488-nm laser for 20 s, and on-switching events were measured for 5 min with 1 s time resolution. Drastic decrement of fluorescence intensity in the first switching cycle is probably due to the insufficient time for the spontaneous transition to the brighter state. **d** Off-switching rates measured in each switching cycle in **c** (displayed first 300 ms of total 20-s of illumination). No notable differences were observed. **e** On-switching rates measured in each switching cycle in **c** (displayed first 100 s of total 300-s of recovery). Slower on-switching rates were observed in later switching cycles due to the local depletion of BR by direct photo-oxidation. Error bars: standard deviations (n = 5 individual measurements for **a**; 3 individual measurements for **b**)..

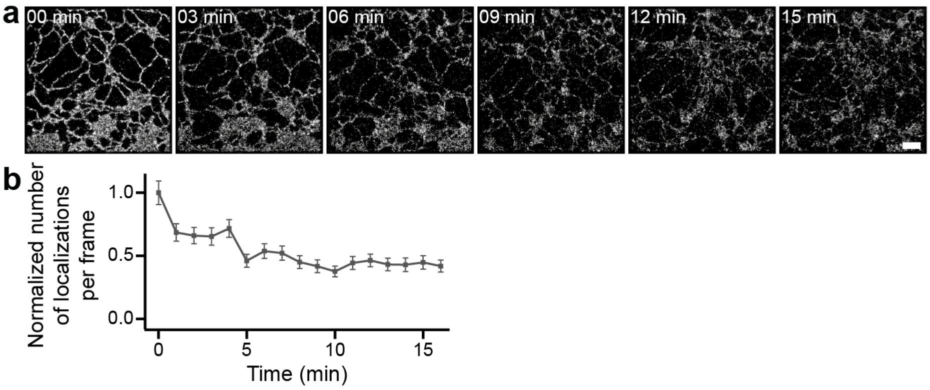

Supplementary Figure 17 Long-term live-cell SML imaging with UnaG. **a** Time-lapse SML snapshots of a UnaG-Sec61β expressing live Cos7 cell from the same dataset used in Fig. 5 and Supplementary Movie 1. The raw data were continuously recorded under SML imaging conditions, and each snapshot 1-s temporal resolution. **b** Time-dependent normalized number of localization per frame. Notable decrement in fast time range came from overactivated acquisition conditions for the fast temporal resolution. Scale bar: 2 μm. Error bars: standard deviations (n = 100 camera frames from each point).

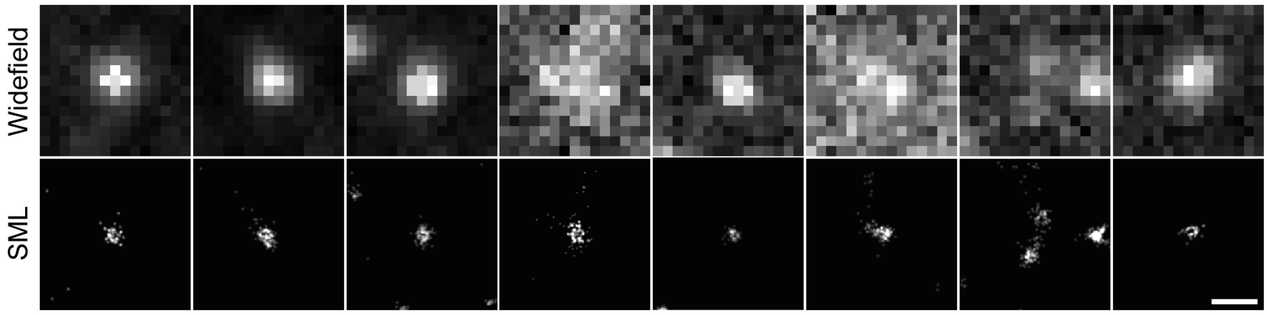

Supplementary Figure 18 2-s snapshots of UnaG-labeled clathrin-coated pits in live Cos7 cells with 10-fold higher excitation intensity. The SML images were reconstructed from 200 frames at 10 ms exposure time that were analyzed with standard single-emitter fitting algorithm without further processing. 1 µM of external bilirubin was supplemented in solution. Increased excitation intensity promoted the off-switching rate, thus reduced the on-off duty cycle by ten times. Under this condition, most of UnaG molecules switched off in a single frame, and sufficiently low duty cycle was achieved for single-emitter fitting algorithm. Scale bar: 500 nm.

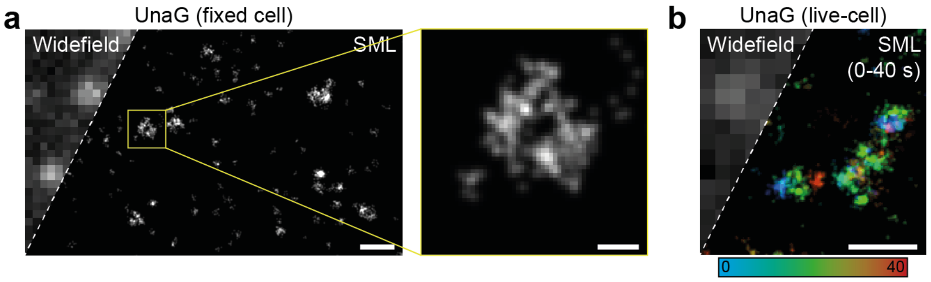

Supplementary Figure 19 SML imaging of clathrin-coated pits (CCP) with UnaG-CLC with endogenous concentration of bilirubin without supplementing bilirubin in the imaging buffer. **a** CCPs in a fixed Cos7 cell whose ring shapes were resolved. The image was reconstructed from 20,000 frames acquired at 10 ms exposure time for total 200 s. **b** CCPs in a live Cos7 cell. The image was reconstructed from 800 frames acquired at 50 ms exposure time. The long acquisition time of total 40 s enough for the pits to move and to change shape in live cells resulted in motion blur. To visualize the movement and evolution of pits, we temporally color-coded the SML image as indicated in the color bar underneath the SML image. In particular, the pit in the lower left corner of the SML image showed color rainbow from blue on the left to red on the right, indicating directional motion from the left to the right. Scale bars: 500 nm for **a** and **b**; 100 nm for the inset in **a**.

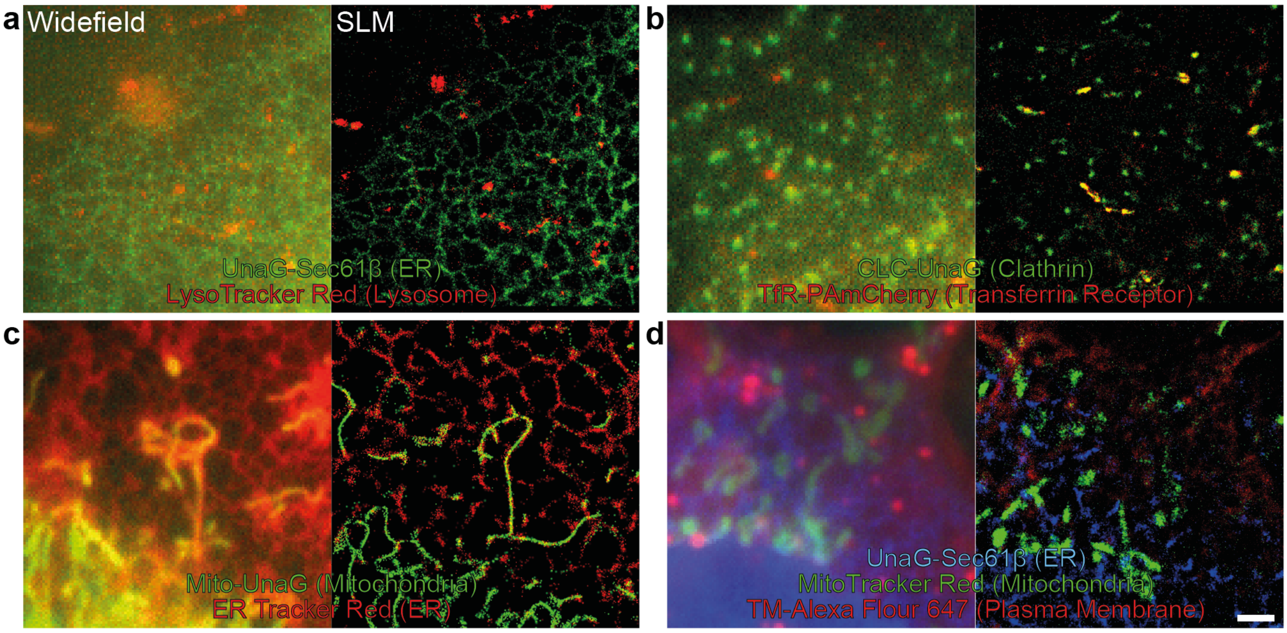

Supplementary Figure 20 Live-cell multicolor SML imaging of UnaG with spectrally distinct probes such as photoswitchable (or photoactivatable) lipophilic dyes (LysoTracker Red, ER Tracker Red, MitoTracker Red), fluorescent protein (PAmCherry) and organic fluorophore (Alexa Fluor 647). **a-c** Examples for two-color live cell SML images of a live Cos7 cell co-stained with **a** UnaG-Sec61β and LysoTracker Red, **b** CLC-UnaG and TfR-PAmCherry, and **c** Mito-UnaG and ER Tracker Red. **d** Demonstration of three-color live-cell SML imaging using UnaG, MitoTracker Red, and Alexa Fluor 647 linked to HaloTag on the plasma membrane. The low crosstalk of blue signals from UnaG into other color channels enables filter-based multicolor SML imaging up to three colors. Scale bar: 2 µm.

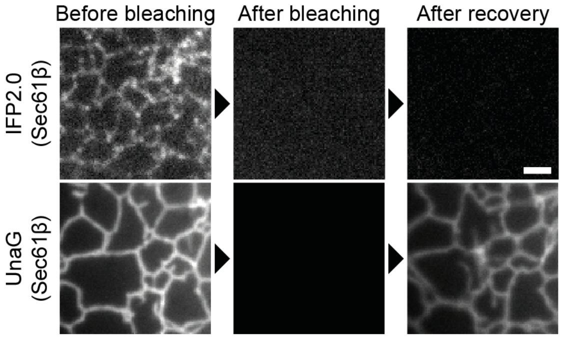

Supplementary Figure 21 Fluorescence switching test of ligand inducible fluorescent proteins, IPF2.0 (top panels) and UnaG (bottom panels). Both proteins were labeled on Sec61β to visualize the endoplasmic reticulum. Photobleaching was carried with 120 mW of 647-mW laser or 150 mW of 488-nm laser for 1 min. To observe the fluorescence recovery, 1 μM of biliverdin or bilirubin were incubated for 10 mins. IFP2.0 did not show any recovered fluorescence probably due to the covalent bond between the ligand and protein. Scale bar: 2 μm.

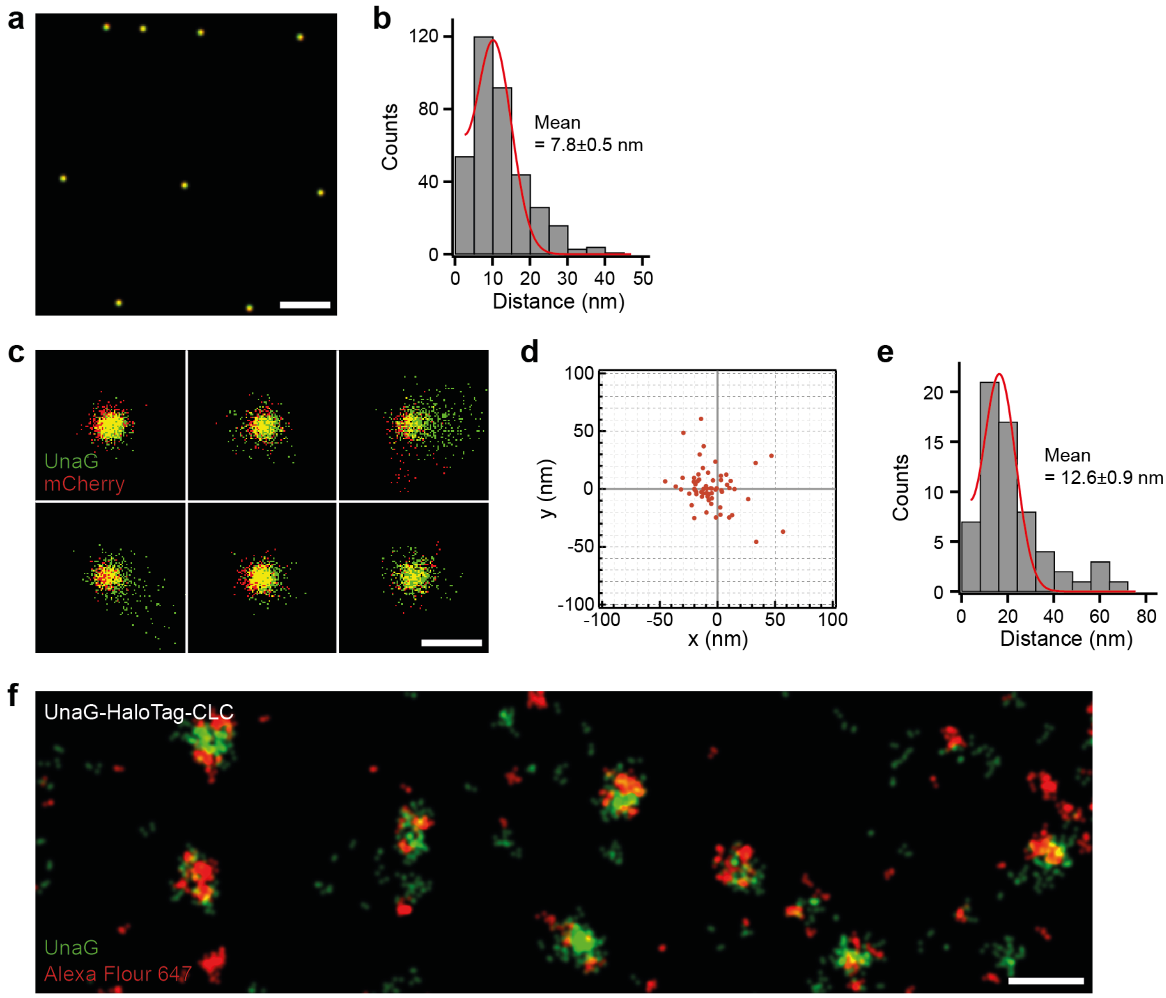

Supplementary Figure 22 Colocalization analysis. **a** Two-channel alignment using fluorescent beads (TetraSpek) whose emission spectrum is spread out from blue to far-red. The centroid positions of the beads in the two emission windows of 500-550 nm and 590-620 nm were localized to align the two channels by spatial transformation. **b** Histogram for the distances between two color channels measured from **a**. Fitting the histogram with normal difference distribution yielded ~8 nm of mean alignment error. **c** Example SML images of surface-tethered UnaG-mCherry complex, displayed as colored scatter dots (Green: UnaG, Red: mCherry) with 2-nm pixel size. **d** Scatter plot for the center-to-center distances between UnaG and mCherry molecules. **e** Histogram for the center-to-center distances displayed with a normal difference distribution fit that yielded ~13 nm of mean distance. **f** Two-color image of a UnaG-HaloTag-CLC expressing fixed Cos7 cell. The HaloTag was labeled with Alexa Fluor 647. Scale bars: 500 nm for **a** and **f**; 20 nm for **c**. Error values (± values): fitting errors for **b** and **e**.
