# Supplementary material for: Bright ligand-activatable fluorescent protein for high-quality multicolor live-cell super-resolution microscopy
Source: Nat Commun. 2020 Jan 14;11:273. doi: 10.1038/s41467-019-14067-4 (PMC6959352; doi:10.1038/s41467-019-14067-4)
Supplement: Supplementary file 3 — Description of Additional Supplementary Files [file 41467_2019_14067_MOESM3_ESM.pdf]

## Description of Additional Supplementary Files

**File name:** Supplementary Movie 1

**Description:** Super resolution movie of the ER in a live Cos7 cell transfected with UnaG-Sec61β. The field of view is the same as that shown in Fig. 5a. The movie runs at 2× real time with 1-s snapshots. Scale bar: 2 μm.
